# Supplementary material for: Novel Giant Phages vB_AerVM_332-Vera and vB_AerVM_332-Igor and Siphophage vB_AerVS_332-Yulya Infecting the Same Aeromonas veronii Strain
Source: Viruses. 2025 Jul 22;17(8):1027. doi: 10.3390/v17081027 (PMC12390700; doi:10.3390/v17081027)
Supplement: Supplementary file 1 [file viruses-17-01027-s001.zip › viruses-3335593-supplementary/Data S1.pdf]

Data S1. Annotation of the Aeromonas phage vB_AerVM_332-Yuliya

#	Feature function	Location	Size (bp)	Directionality
1.	terminase small subunit	182..625	444	=>
2.	hypothetical protein	627..827	201	=>
3.	terminase large subunit	824..2587	1764	=>
4.	portal protein	2599..3879	1281	=>
5.	prohead protease	3869..4516	648	=>
6.	major capsid protein	4603..5976	1374	=>
7.	hypothetical protein	6038..6277	240	=>
8.	head-tail adaptor	6374..6883	510	=>
9.	stopper protein	6880..7212	333	=>
10.	hypothetical protein	7205..8017	813	=>
11.	tail terminator	8014..8367	354	=>
12.	tail tube protein	8379..9068	690	=>
13.	tail assembly chaperone	9065..9436	372	=>
14.	hypothetical protein	9448..9648	201	=>
15.	neck protein	9648..10,175	528	=>
16.	tape measure protein	10,180..12,579	2400	=>
17.	distal tail protein	12,579..13,073	495	=>
18.	hypothetical protein	13,073..13,588	516	=>
19.	peptidoglycan endopeptidase	13,585..13,941	357	=>
20.	baseplate protein	13,908..16,640	2733	=>
21.	hypothetical protein	16,670..16,864	195	<=
22.	hypothetical protein	16,864..17,172	309	<=
23.	DNA polymerase	17,172..19,109	1938	<=
24.	hypothetical protein	19,244..19,513	270	<=
25.	hypothetical protein	19,572..19,706	135	<=
26.	hypothetical protein	19,703..19,885	183	<=
27.	hypothetical protein	19,882..20,112	231	<=
28.	hypothetical protein	20,117..20,251	135	<=
29.	hypothetical protein	20,248..20,451	204	<=
30.	DNA primase	20,538..22,991	2454	<=
31.	transcriptional regulator	23,002..23,223	222	<=
32.	holliday junction resolvase	23,220..23,654	435	<=
33.	hypothetical protein	23,647..23,835	189	<=
34.	ssDNA-binding protein	23,891..24,937	1047	<=
35.	hypothetical protein	24,962..25,132	171	<=
36.	exonuclease	25,136..26,203	1068	<=
37.	NAD/FAD-utilizing enzyme	26,287..26,589	303	<=
38.	hypothetical protein	26,652..27,542	891	<=
39.	hypothetical protein	27,668..27,847	180	=>
40.	hypothetical protein	27,828..28,007	180	=>
41.	helicase	28,004..29,794	1791	=>
42.	endolysin	29,890..30,402	513	=>
43.	hypothetical protein	30,392..30,580	189	=>
44.	hypothetical protein	30,584..30,712	129	=>
45.	TMhelix containing protein	30,725..31,228	504	=>
46.	tailspike protein	31,279..33,477	2199	<=
47.	hypothetical protein	33,479..33,763	285	<=
48.	hypothetical protein	33,753..34,064	312	<=
49.	Phage ABA sandwich domain-containing protein	34,114..34,494	381	<=
50.	hypothetical protein	34,491..34,823	333	<=
51.	ParB/Sulfiredoxin domain-containing proteini	34,820..35,635	816	<=
52.	hypothetical protein	35,687..36,025	339	<=

53.	hypothetical protein	36,096..36,356	261	<=
54.	hypothetical protein	36,417..36,530	114	<=
55.	hypothetical protein	36,564..36,737	174	<=
56.	Deoxyuridine 5'-triphosphate nucleotidohydrolase	36,805..37,344	540	<=
57.	transcriptional regulator	37,341..37,625	285	<=
58.	HNH endonuclease	37,687..38,319	633	<=
59.	transcriptional regulator (NtrC family)	38,316..38,702	387	<=
60.	hypothetical protein	38,699..39,406	708	<=
61.	hypothetical protein	39,449..39,631	183	<=
62.	transcriptional regulator	39,632..39,976	345	<=
63.	DNA methyltransferase	39,973..40,671	699	<=
64.	hypothetical protein	40,668..41,186	519	<=
65.	hypothetical protein	41,173..42,075	903	<=
66.	hypothetical protein	42,072..42,236	165	<=
67.	hypothetical protein	42,709..42,891	183	=>
68.	HNH endonuclease	42,881..43,276	396	=>
