# Supplementary material for: Novel Giant Phages vB_AerVM_332-Vera and vB_AerVM_332-Igor and Siphophage vB_AerVS_332-Yulya Infecting the Same Aeromonas veronii Strain
Source: Viruses. 2025 Jul 22;17(8):1027. doi: 10.3390/v17081027 (PMC12390700; doi:10.3390/v17081027)
Supplement: Supplementary file 1 [file viruses-17-01027-s001.zip › viruses-3335593-supplementary/Data S2.pdf]

Data S2. Annotation of the Aeromonas phage vB_AerVM_332-Vera

#	Feature function	Location	Size (bp)	Directionality
1.	RNA polymerase beta subunit	127..792	666	<=
2.	DUF4326 domain-containing protein	756..1280	525	<=
3.	hypothetical protein	1166..1489	324	<=
4.	UvsX-like recombinase	1505..2956	1452	<=
5.	putative ADP-ribose pyrophosphatase	3034..4185	1152	=>
6.	hypothetical protein	4231..4617	387	<=
7.	Ribonuclease HI	4663..6384	1722	<=
8.	hypothetical protein	6425..6910	486	<=
9.	hypothetical protein	6921..7511	591	<=
10.	virion structural protein	7553..8992	1440	=>
11.	baseplate wedge	9023..9622	600	=>
12.	capsid maturation protease	9655..10,560	906	<=
13.	hypothetical protein	10,570..11,172	603	<=
14.	portal protein	11,265..13,739	2475	=>
15.	hypothetical protein	13,736..14,203	468	=>
16.	hypothetical protein	14,200..15,300	1101	=>
17.	virion structural protein	15,333..16,583	1251	<=
18.	tail fiber protein	16,674..19,235	2562	=>
19.	hypothetical protein	19,272..19,628	357	=>
20.	hypothetical protein	19,631..20,086	456	=>
21.	virion structural protein	20,141..21,397	1257	=>
22.	tail fiber domain-containing protein	21,399..21,971	573	=>
23.	virion structural protein;	21,981..23,294	1314	=>
24.	hypothetical protein	23,334..23,696	363	=>
25.	tail fiber protein	23,697..25,499	1803	=>
26.	tail fiber protein	25,509..27,155	1647	=>
27.	cupin domain-containing protein	27,233..27,772	540	=>
28.	radical SAM protein	27,757..28,782	1026	=>
29.	hypothetical protein	28,787..29,332	546	=>
30.	hypothetical protein	29,329..30,048	720	=>
31.	hypothetical protein	30,032..31,021	990	=>
32.	radical SAM protein	31,073..32,080	1008	=>
33.	radical SAM protein	32,084..33,160	1077	=>
34.	radical SAM protein	33,157..34,545	1389	=>
35.	radical SAM protein	34,517..35,623	1107	=>
36.	tail fiber domain-containing protein	35,694..37,334	1641	=>
37.	radical SAM protein	37,386..38,279	894	=>
38.	radical SAM protein	38,251..39,366	1116	=>
39.	hypothetical protein	39,353..39,874	522	=>
40.	keratin associated protein	39,884..40,393	510	=>
41.	keratin associated protein	40,409..40,966	558	=>
42.	radical SAM protein	41,215..42,051	837	=>
43.	hypothetical protein	42,044..42,904	861	=>
44.	hypothetical protein	42,918..43,571	654	=>
45.	radical SAM protein	43,571..44,785	1215	=>
46.	tail assembly-like protein	44,827..45,213	387	=>
47.	lysozyme	45,210..45,848	639	=>
48.	hypothetical protein	45,907..46,350	444	<=
49.	hypothetical protein	46,438..46,617	180	<=
50.	hypothetical protein	46,598..47,347	750	<=
51.	hypothetical protein	47,426..47,641	216	<=
52.	HNH endonuclease	47,743..48,807	1065	<=
53.	hypothetical protein	48,925..49,824	900	<=
54.	hypothetical protein	49,821..50,612	792	<=

55.	dCTP deaminase	50,663..51,343	681	<=
56.	hypothetical protein	51,353..52,060	708	<=
57.	hypothetical protein	52,053..52,640	588	<=
58.	hypothetical protein	52,681..53,316	636	<=
59.	Deoxyuridine 5'-triphosphate nucleotidohydrolase	53,370..53,834	465	<=
60.	hypothetical protein	53,768..54,397	630	<=
61.	hypothetical protein	54,434..55,393	960	<=
62.	hypothetical protein	55,393..55,935	543	<=
63.	hypothetical protein	56,065..58,095	2031	<=
64.	hypothetical protein	58,098..58,334	237	<=
65.	DEAD-like helicase	58,344..60,437	2094	<=
66.	hypothetical protein	60,527..61,213	687	<=
67.	hypothetical protein	61,226..61,909	684	<=
68.	hypothetical protein	61,906..62,370	465	<=
69.	hypothetical protein	62,348..63,010	663	<=
70.	hypothetical protein	63,035..63,466	432	<=
71.	dihydrofolate reductase	63,459..64,097	639	<=
72.	phosphoesterase	64,107..64,700	594	<=
73.	hypothetical protein	64,697..65,155	459	<=
74.	hypothetical protein	65,118..65,792	675	<=
75.	hypothetical protein	65,770..66,249	480	<=
76.	hypothetical protein	66,251..66,607	357	<=
77.	hypothetical protein	66,604..66,939	336	<=
78.	hypothetical protein	66,990..68,225	1236	<=
79.	hypothetical protein	68,350..68,778	429	<=
80.	hypothetical protein	68,768..69,181	414	<=
81.	hypothetical protein	69,192..69,719	528	<=
82.	hypothetical protein	69,950..70,699	750	<=
83.	hypothetical protein	70,699..72,129	1431	<=
84.	hypothetical protein	72,178..72,639	462	<=
85.	hypothetical protein	72,644..73,045	402	<=
86.	hypothetical protein	73,097..73,762	666	<=
87.	hypothetical protein	73,766..74,134	369	<=
88.	hypothetical protein	74,240..74,359	120	<=
89.	hypothetical protein	74,372..74,482	111	<=
90.	hypothetical protein	74,492..74,596	105	<=
91.	hypothetical protein	74,593..74,706	114	<=
92.	hypothetical protein	74,836..75,702	867	<=
93.	hypothetical protein	75,808..76,521	714	<=
94.	hypothetical protein	76,631..77,653	1023	<=
95.	hypothetical protein	77,657..78,184	528	<=
96.	hypothetical protein	78,241..79,245	1005	<=
97.	hypothetical protein	79,349..80,404	1056	<=
98.	hypothetical protein	80,459..80,905	447	<=
99.	hypothetical protein	80,963..81,268	306	<=
100.	PnuC-like nicotinamide mononucleotide transport	81,289..82,047	759	<=
101.	nicotinamide-nucleotide adenylyltransferase, Na	82,092..84,581	2490	<=
102.	hypothetical protein	84,631..85,539	909	<=
103.	hypothetical protein	85,589..86,269	681	<=
104.	hypothetical protein	86,273..86,554	282	<=
105.	hypothetical protein	86,573..87,427	855	<=
106.	DUF1874 domain-containing protein	87,497..87,991	495	<=
107.	hypothetical protein	88,145..89,836	1692	<=
108.	hypothetical protein	89,892..90,344	453	<=

109.	hypothetical protein	90,354..91,100	747	<=
110.	DNA ligase	91,159..93,303	2145	<=
111.	hypothetical protein	93,300..93,767	468	<=
112.	hypothetical protein	93,754..94,332	579	<=
113.	hypothetical protein	94,401..94,985	585	<=
114.	hypothetical protein	95,038..95,346	309	<=
115.	hypothetical protein	95,356..95,778	423	<=
116.	hypothetical protein	95,871..96,083	213	<=
117.	hypothetical protein	96,091..96,894	804	<=
118.	hypothetical protein	96,945..98,039	1095	<=
119.	hypothetical protein	98,053..98,646	594	<=
120.	putative hydrolase	98,639..99,427	789	<=
121.	hypothetical protein	99,534..99,926	393	<=
122.	hypothetical protein	99,938..100,777	840	<=
123.	hypothetical protein	100,902..102,041	1140	<=
124.	hypothetical protein	102,095..102,676	582	<=
125.	hypothetical protein	102,676..103,839	1164	<=
126.	hypothetical protein	103,839..104,744	906	<=
127.	hypothetical protein	104,744..105,451	708	<=
128.	hypothetical protein	105,445..106,395	951	<=
129.	hypothetical protein	106,399..107,433	1035	<=
130.	hypothetical protein	107,482..107,760	279	<=
131.	hypothetical protein	107,805..108,047	243	<=
132.	hypothetical protein	108,127..108,579	453	<=
133.	hypothetical protein	108,581..108,886	306	<=
134.	hypothetical protein	108,898..109,083	186	<=
135.	hypothetical protein	109,135..109,911	777	<=
136.	hypothetical protein	109,908..110,432	525	<=
137.	hypothetical protein	110,445..111,452	1008	<=
138.	hypothetical protein	111,439..111,756	318	<=
139.	hypothetical protein	111,749..112,078	330	<=
140.	hypothetical protein	112,068..112,742	675	<=
141.	hypothetical protein	112,735..113,157	423	<=
142.	hypothetical protein	113,150..113,746	597	<=
143.	hypothetical protein	113,730..114,149	420	<=
144.	hypothetical protein	114,139..114,807	669	<=
145.	hypothetical protein	114,811..115,737	927	<=
146.	hypothetical protein	115,833..116,558	726	<=
147.	hypothetical protein	116,570..116,938	369	<=
148.	hypothetical protein	116,970..117,353	384	<=
149.	transglycosylase	117,432..118,055	624	<=
150.	hypothetical protein	118,180..118,884	705	<=
151.	hypothetical protein	118,865..119,365	501	<=
152.	hypothetical protein	119,375..120,379	1005	<=
153.	hypothetical protein	120,379..121,485	1107	<=
154.	hypothetical protein	121,501..122,202	702	<=
155.	hypothetical protein	122,314..123,156	843	<=
156.	hypothetical protein	123,179..123,433	255	<=
157.	hypothetical protein	123,460..123,759	300	<=
158.	hypothetical protein	123,765..124,028	264	<=
159.	thymidylate synthase	124,074..125,276	1203	<=
160.	hypothetical protein	125,363..125,560	198	<=
161.	hypothetical protein	125,563..126,627	1065	<=
162.	hypothetical protein	126,627..126,965	339	<=
163.	hypothetical protein	127,057..127,644	588	<=
164.	thymidylate kinase	127,626..128,294	669	<=
165.	holliday junction resolvase	128,278..128,862	585	<=

166.	virion structural protein	128,862..129,737	876	<=
167.	GNAT family N-acetyltransferase	129,804..130,289	486	<=
168.	hypothetical protein	130,369..131,715	1347	<=
169.	virion structural protein	131,777..132,694	918	<=
170.	hypothetical protein	132,767..136,696	3930	<=
171.	hypothetical protein	136,696..138,249	1554	<=
172.	hypothetical protein	138,260..139,252	993	<=
173.	hypothetical protein	139,249..140,214	966	<=
174.	virion structural protein	140,257..141,582	1326	<=
175.	virion structural protein	141,595..142,434	840	<=
176.	virion structural protein	142,438..143,346	909	<=
177.	hypothetical protein	143,357..144,616	1260	<=
178.	virion structural protein	144,603..145,505	903	<=
179.	virion structural protein	145,515..146,348	834	<=
180.	virion structural protein	146,447..146,941	495	<=
181.	virion structural protein	146,989..147,483	495	<=
182.	virion structural protein	147,523..148,362	840	<=
183.	virion structural protein	148,373..149,200	828	<=
184.	virion structural protein	149,200..150,033	834	<=
185.	hypothetical protein	150,044..150,949	906	<=
186.	hypothetical protein	150,959..151,873	915	<=
187.	virion structural protein	151,882..152,709	828	<=
188.	virion structural protein	152,709..153,665	957	<=
189.	virion structural protein	153,731..155,821	2091	<=
190.	virion structural protein	155,849..158,659	2811	=>
191.	hypothetical protein	158,726..159,712	987	<=
192.	hypothetical protein	159,725..160,624	900	<=
193.	hypothetical protein	160,673..162,766	2094	<=
194.	RNA polymerase, non-virion	162,789..164,399	1611	<=
195.	Virion structural protein	164,409..165,770	1362	<=
196.	major capsid protein	165,841..168,045	2205	<=
197.	hypothetical protein	168,118..168,687	570	<=
198.	DnaB-like replicative helicase	168,737..170,269	1533	=>
199.	hypothetical protein	170,367..170,777	411	<=
200.	hypothetical protein	170,793..170,996	204	<=
201.	hypothetical protein	171,143..172,300	1158	<=
202.	hypothetical protein	172,589..172,987	399	<=
203.	virion structural protein	173,055..174,446	1392	<=
204.	hypothetical protein	174,437..175,024	588	<=
205.	virion structural protein	175,024..176,406	1383	<=
206.	hypothetical protein	176,416..176,733	318	<=
207.	hypothetical protein	176,730..178,235	1506	<=
208.	hypothetical protein	178,410..180,185	1776	<=
209.	hypothetical protein	180,195..181,187	993	<=
210.	hypothetical protein	181,265..182,353	1089	<=
211.	hypothetical protein	182,412..183,611	1200	<=
212.	hypothetical protein	183,639..185,090	1452	<=
213.	hypothetical protein	185,186..186,340	1155	<=
214.	hypothetical protein	186,389..187,576	1188	<=
215.	virion structural protein	187,579..188,052	474	<=
216.	virion structural protein	188,036..188,950	915	<=
217.	virion structural protein	188,965..190,257	1293	<=
218.	virion structural protein	190,289..191,368	1080	=>
219.	virion structural protein	191,380..194,289	2910	=>
220.	hypothetical protein	194,350..195,822	1473	<=
221.	hypothetical protein	195,812..196,912	1101	<=
222.	hypothetical protein	196,915..197,406	492	<=

223.	membrane-bound metallopeptidase	197,418..198,710	1293	<=
224.	Ribonuclease H	198,828..200,564	1737	=>
225.	hypothetical protein	200,602..203,949	3348	<=
226.	hypothetical protein	204,007..204,630	624	<=
227.	RNA polymerase beta subunit	204,640..205,950	1311	<=
228.	hypothetical protein	205,952..206,785	834	<=
229.	hypothetical protein	206,798..207,286	489	=>
230.	baseplate protein	207,286..208,881	1596	=>
231.	hypothetical protein	208,929..209,288	360	=>
232.	RNase III inhibitor, macrodomain protein	209,298..209,735	438	=>
233.	hypothetical protein	209,792..210,244	453	<=
234.	hypothetical protein	210,241..210,348	108	<=
235.	hypothetical protein	210,345..211,010	666	<=
236.	hypothetical protein	211,133..211,495	363	<=
237.	DUF4262 domain-containing protein	211,467..212,012	546	<=
238.	hypothetical protein	212,025..212,519	495	<=
239.	DEAD/DEAH box helicase protein	212,620..214,143	1524	<=
240.	RNA polymerase subunit beta'	214,159..216,240	2082	<=
241.	DNA-directed RNA polymerase subunit alpha	216,243..218,336	2094	<=
242.	baseplate wedge protein	218,410..218,742	333	=>
243.	hypothetical protein	218,836..220,311	1476	<=
244.	hypothetical protein	220,289..221,770	1482	<=
245.	RNA polymerase, non-virion	221,791..223,338	1548	<=
246.	Single-stranded DNA-binding protein	223,440..224,243	804	<=
247.	hypothetical protein	224,300..225,121	822	<=
248.	nuclease SbcCD subunit D	225,118..226,236	1119	<=
249.	hypothetical protein	226,217..226,774	558	<=
250.	hypothetical protein	226,780..227,124	345	<=
251.	hypothetical protein	227,229..227,669	441	<=
252.	hypothetical protein	227,666..227,968	303	<=
253.	hypothetical protein	227,978..228,316	339	<=
254.	hypothetical protein	228,309..228,542	234	<=
255.	hypothetical protein	228,586..229,197	612	<=
256.	hypothetical protein	229,291..229,671	381	<=
257.	RNA polymerase subunit beta/beta'	229,704..231,281	1578	<=
258.	nuclear shell protein	231,367..233,223	1857	<=
259.	hypothetical protein	233,440..234,540	1101	=>
260.	hypothetical protein	234,576..235,619	1044	<=
261.	DNA polymerase B	235,629..237,767	2139	<=
262.	phage DNA polymerase-associated SH3 family protein	237,854..238,201	348	<=
263.	hypothetical protein	238,218..238,682	465	<=
264.	hypothetical protein	239,082..239,522	441	<=
265.	hypothetical protein	239,545..240,006	462	<=
266.	hypothetical protein	239,994..240,971	978	<=
267.	hypothetical protein	240,974..241,294	321	<=
268.	hypothetical protein	241,742..241,984	243	<=
269.	hypothetical protein	241,996..242,385	390	<=
270.	tubulin	242,434..243,426	993	<=
271.	hypothetical protein	243,419..243,748	330	<=
272.	virion structural protein	243,823..244,662	840	=>
273.	hypothetical protein	244,693..245,184	492	<=
274.	hypothetical protein	245,452..246,204	753	<=
275.	hypothetical protein	246,217..247,497	1281	<=
276.	hypothetical protein	247,546..248,361	816	<=

277.	hypothetical protein	248,413..249,294	882	<=
278.	putative nuclease	249,346..249,897	552	<=
279.	hypothetical protein	250,323..250,733	411	<=
280.	hypothetical protein	250,743..251,330	588	<=
281.	hypothetical protein	251,439..251,792	354	<=
282.	hypothetical protein	251,892..252,179	288	<=
283.	hypothetical protein	252,216..253,028	813	<=
284.	virion-associated head protein	253,092..254,471	1380	=>
285.	terminase large subunit	254,532..256,754	2223	<=
286.	virion structural protein	256,809..258,485	1677	<=
287.	virion structural protein	258,492..261,041	2550	<=
288.	virion structural protein	261,059..261,979	921	<=
289.	tail sheath protein	262,113..264,155	2043	=>
290.	virion structural protein	264,152..265,015	864	=>
291.	hypothetical protein	265,082..265,777	696	<=
292.	hypothetical protein	265,827..266,390	564	<=
293.	hypothetical protein	266,521..266,949	429	<=
294.	NADAR domain-containing protein	266,959..267,513	555	<=
295.	GIY-YIG nuclease family protein	267,513..267,896	384	<=
296.	hypothetical protein	267,884..268,135	252	<=
297.	hypothetical protein	268,201..268,473	273	<=
298.	hypothetical protein	268,536..269,018	483	<=
299.	head maturation protease	269,199..269,966	768	<=
300.	hypothetical protein	269,976..270,674	699	<=
301.	virion structural protein	270,674..271,393	720	<=
302.	hypothetical protein	271,438..272,955	1518	<=
303.	hypothetical protein	272,968..273,150	183	<=
304.	RNA polymerase beta subunit	273,147..277,520	4374	<=
305.	RNA polymerase beta subunit	277,513..279,153	1641	<=
306.	transglycosylase domain-containing tail fiber prot	279,214..285,897	6684	=>
307.	virion structural protein	285,946..287,970	2025	=>
308.	virion structural protein	288,017..289,036	1020	<=
309.	hypothetical protein	289,109..289,549	441	<=
310.	hypothetical protein	289,610..290,398	789	<=
311.	hypothetical protein	290,523..291,068	546	<=
312.	hypothetical protein	291,080..291,913	834	<=
313.	hypothetical protein	291,966..292,364	399	<=
314.	hypothetical protein	292,364..292,870	507	<=
315.	hypothetical protein	292,954..293,406	453	=>
316.	hypothetical protein	293,446..294,150	705	<=
317.	hypothetical protein	294,140..294,685	546	<=
