# Supplementary material for: Novel Giant Phages vB_AerVM_332-Vera and vB_AerVM_332-Igor and Siphophage vB_AerVS_332-Yulya Infecting the Same Aeromonas veronii Strain
Source: Viruses. 2025 Jul 22;17(8):1027. doi: 10.3390/v17081027 (PMC12390700; doi:10.3390/v17081027)
Supplement: Supplementary file 1 [file viruses-17-01027-s001.zip › viruses-3335593-supplementary/Data S3.pdf]

Data S3. Annotation of the Aeromonas phage vB_AerVM_332-Igor

#	Feature function	Location	Size (bp)	Directionality
1.	hypothetical protein	2..331	330	=>
2.	polyVal ADP-ribosyltransferase	550..1224	675	=>
3.	hypothetical protein	1250..1423	174	=>
4.	nudix hydrolase	1436..1870	435	=>
5.	mutT/NUDIX hydrolase	1877..2794	918	=>
6.	hypothetical protein	2805..3323	519	=>
7.	poly [ADP-ribose] polymerase 1	3375..6152	2778	=>
8.	hypothetical protein	6171..6371	201	=>
9.	hypothetical protein	6381..7214	834	=>
10.	hypothetical protein	7214..8143	930	=>
11.	hypothetical protein	8153..8995	843	=>
12.	hypothetical protein	9082..10,476	1395	=>
13.	hypothetical protein	10,487..11,023	537	=>
14.	hypothetical protein	11,013..11,624	612	=>
15.	phosphoglycolate phosphatase	11,621..12,364	744	=>
16.	hydrolase	12,375..13,361	987	=>
17.	hypothetical protein	13,358..13,699	342	=>
18.	AAA+ class ATPase [	13,712..15,181	1470	=>
19.	hypothetical protein	15,268..15,684	417	=>
20.	lysozyme	15,684..16,181	498	=>
21.	tail sheath protein	16,178..17,836	1659	=>
22.	structural tail protein	17,849..18,481	633	=>
23.	long tail fiber protein	18,484..19,104	621	=>
24.	tail protein	19,109..23,047	3939	=>
25.	hypothetical protein	23,065..23,856	792	=>
26.	tail sheath	23,860..24,807	948	=>
27.	tail sheath	24,822..25,985	1164	=>
28.	virion structural protein	26,059..29,877	3819	=>
29.	putative metallopeptidase	29,932..32,304	2373	=>
30.	NAD-dependent DNA ligase	32,342..34,306	1965	=>
31.	hypothetical protein	34,303..35,199	897	=>
32.	beta-1,4-glycosyltransferase	35,196..36,359	1164	=>
33.	hypothetical protein	36,346..37,479	1134	=>
34.	head maturation protease	37,483..38,274	792	=>
35.	peptidase	38,274..42,515	4242	=>
36.	ATPase	42,557..44,821	2265	=>
37.	virion structural protein	44,907..45,605	699	=>
38.	major head protein	45,690..46,772	1083	=>
39.	hypothetical protein	46,769..47,203	435	=>
40.	hypothetical protein	47,263..48,063	801	=>
41.	hypothetical protein	48,066..48,479	414	=>
42.	tail sheath	48,493..50,202	1710	=>
43.	hypothetical protein	50,199..50,939	741	=>
44.	tail tube protein	50,946..51,464	519	=>
45.	hypothetical protein	51,501..52,154	654	=>
46.	tail tube protein	52,151..52,660	510	=>
47.	hypothetical protein	52,663..53,400	738	=>
48.	baseplate hub assembly protein	53,397..54,266	870	=>
49.	lytic transglycosylase domain-containing protein	54,266..56,827	2562	=>
50.	tail assembly protein	56,827..57,627	801	=>
51.	tail sheath	57,637..57,906	270	=>
52.	hypothetical protein	57,919..59,757	1839	=>
53.	baseplate hub subunit	59,759..60,580	822	=>
54.	hypothetical protein	60,590..61,423	834	=>

55.	hypothetical protein	61,461..61,835	375	=>
56.	DUF1073 domain-containing protein	61,845..62,396	552	=>
57.	MmcB-like DNA repair protein	62,461..62,868	408	=>
58.	hypothetical protein	62,865..63,560	696	=>
59.	hypothetical protein	63,564..64,238	675	=>
60.	hypothetical protein	64,238..64,603	366	=>
61.	thymidylate kinase	64,600..65,250	651	=>
62.	hypothetical protein	65,260..66,783	1524	=>
63.	peptidase M15A	66,825..67,319	495	<=
64.	dCMP deaminase	67,371..67,916	546	=>
65.	Thymidylate synthase	67,913..69,259	1347	=>
66.	hypothetical protein	69,262..69,822	561	=>
67.	hypothetical protein	69,819..70,196	378	=>
68.	hypothetical protein	70,187..70,684	498	=>
69.	hypothetical protein	70,771..71,202	432	=>
70.	hypothetical protein	71,199..72,002	804	=>
71.	hypothetical protein	71,995..72,474	480	=>
72.	hypothetical protein	72,477..72,767	291	=>
73.	hypothetical protein	72,764..73,024	261	=>
74.	dihydrofolate reductase	73,026..73,685	660	=>
75.	lipoprotein	73,723..74,208	486	<=
76.	hypothetical protein	74,216..74,503	288	<=
77.	hypothetical protein	74,500..74,811	312	<=
78.	hypothetical protein	74,828..75,070	243	<=
79.	hypothetical protein	75,114..75,608	495	<=
80.	hypothetical protein	75,620..76,105	486	<=
81.	hypothetical protein	76,107..76,586	480	<=
82.	hypothetical protein	76,588..77,076	489	<=
83.	phage tail protein	77,128..77,685	558	<=
84.	hypothetical protein	77,723..78,253	531	<=
85.	hypothetical protein	78,258..79,292	1035	<=
86.	hypothetical protein	79,305..79,532	228	<=
87.	hypothetical protein	79,543..80,184	642	<=
88.	hypothetical protein	80,194..80,937	744	<=
89.	hypothetical protein	80,948..81,529	582	<=
90.	hypothetical protein	81,531..82,070	540	<=
91.	hypothetical protein	82,074..82,622	549	<=
92.	hypothetical protein	82,619..83,215	597	<=
93.	hypothetical protein	83,225..83,722	498	<=
94.	hypothetical protein	83,734..84,171	438	<=
95.	hypothetical protein	84,181..84,645	465	<=
96.	hypothetical protein	84,690..84,923	234	<=
97.	hypothetical protein	84,938..85,555	618	<=
98.	virion structural protein	85,622..87,319	1698	<=
99.	ABC transporter	87,331..88,074	744	<=
100.	hypothetical protein	88,071..88,550	480	<=
101.	hypothetical protein	88,550..88,843	294	<=
102.	hypothetical protein	88,857..89,642	786	<=
103.	HNH endonuclease	89,657..90,439	783	<=
104.	tail fiber protein	90,442..91,263	822	<=
105.	hypothetical protein	91,277..91,498	222	<=
106.	long tail fiber protein	91,498..95,754	4257	<=
107.	large intein domain protein	95,764..97,848	2085	<=
108.	tail fiber protein	97,858..98,373	516	<=
109.	tail fiber protein	98,384..99,004	621	<=
110.	virion structural protein	99,004..99,954	951	<=
111.	tail-collar fiber protein	99,959..102,316	2358	<=

112.	tail protein	102,348..107,096	4749	<=
113.	base-plate wedget subunit	107,083..108,540	1458	<=
114.	baseplate protein	108,542..108,973	432	<=
115.	PAAR motif of membran proteins	108,975..109,268	294	<=
116.	hypothetical protein	109,343..110,050	708	<=
117.	hypothetical protein	110,305..110,907	603	<=
118.	hypothetical protein	110,909..111,820	912	<=
119.	DNA polymerase I (5'-3' exonuclease domain)	111,824..112,762	939	<=
120.	hypothetical protein	112,759..113,208	450	<=
121.	hypothetical protein	113,205..113,411	207	<=
122.	hypothetical protein	113,431..113,823	393	<=
123.	hypothetical protein	113,828..114,112	285	<=
124.	hypothetical protein	114,109..114,756	648	<=
125.	portal protein	114,767..116,701	1935	<=
126.	DNA ligase	116,706..116,951	246	<=
127.	terminase large subunit	116,948..120,118	3171	<=
128.	hypothetical protein	120,126..120,890	765	<=
129.	DnaB-like replicative helicase	120,871..122,382	1512	<=
130.	hypothetical protein	122,391..122,732	342	<=
131.	hypothetical protein	122,729..123,844	1116	<=
132.	DNA topoisomerase	123,868..124,752	885	<=
133.	putative HNH endonuclease	124,739..125,134	396	<=
134.	hypothetical protein	125,264..126,751	1488	<=
135.	DNA topoisomerase II	126,899..128,629	1731	<=
136.	DNA gyrase subunit B	128,633..130,645	2013	<=
137.	hypothetical protein	130,647..130,988	342	<=
138.	hypothetical protein	131,081..131,770	690	<=
139.	hypothetical protein	131,852..132,457	606	<=
140.	phage protein	132,605..133,555	951	<=
141.	hypothetical protein	133,665..134,198	534	<=
142.	hypothetical protein	134,264..134,377	114	<=
143.	hypothetical protein	134,378..134,815	438	<=
144.	hypothetical protein	134,875..138,048	3174	<=
145.	hypothetical protein	138,061..139,287	1227	<=
146.	hypothetical protein	139,291..139,728	438	<=
147.	hypothetical protein	139,766..140,128	363	<=
148.	hypothetical protein	140,184..141,533	1350	<=
149.	hypothetical protein	141,758..142,381	624	<=
150.	hypothetical protein	142,443..143,048	606	<=
151.	hypothetical protein	143,157..143,765	609	<=
152.	hypothetical protein	143,808..144,434	627	<=
153.	hypothetical protein	144,447..144,893	447	<=
154.	hypothetical protein	144,988..145,518	531	<=
155.	hypothetical protein	145,515..146,426	912	<=
156.	hypothetical protein	146,428..146,748	321	<=
157.	hypothetical protein	146,758..146,949	192	<=
158.	hypothetical protein	146,957..147,322	366	<=
159.	hypothetical protein	147,325..147,858	534	<=
160.	hypothetical protein	147,908..149,464	1557	<=
161.	hypothetical protein	149,514..149,795	282	<=
162.	hypothetical protein	149,797..150,291	495	<=
163.	hypothetical protein	150,300..150,782	483	<=
164.	hypothetical protein	150,834..151,109	276	<=
165.	hypothetical protein	151,174..151,395	222	<=
166.	hypothetical protein	151,392..151,550	159	<=
167.	hypothetical protein	151,562..152,707	1146	<=

168.	hypothetical protein	152,757..153,251	495	<=
169.	hypothetical protein	153,251..153,727	477	<=
170.	dUTPase	153,727..154,593	867	<=
171.	hypothetical protein	154,657..155,223	567	<=
172.	hypothetical protein	155,232..155,879	648	<=
173.	hypothetical protein	155,881..156,246	366	<=
174.	NrdC-like thioredoxin	156,264..156,509	246	<=
175.	transglycosylase	156,574..157,173	600	<=
176.	hypothetical protein	157,255..157,533	279	<=
177.	hypothetical protein	157,616..157,993	378	<=
178.	hypothetical protein	158,044..158,736	693	<=
179.	hypothetical protein	158,729..158,992	264	<=
180.	hypothetical protein	159,002..159,202	201	<=
181.	hypothetical protein	159,242..159,787	546	<=
182.	hypothetical protein	159,903..160,373	471	<=
183.	hypothetical protein	160,376..160,867	492	<=
184.	hypothetical protein	160,867..161,421	555	<=
185.	hypothetical protein	161,432..162,064	633	<=
186.	hypothetical protein	162,412..163,143	732	<=
187.	transcriptional regulator	163,153..163,584	432	<=
188.	hypothetical protein	163,665..164,243	579	<=
189.	hypothetical protein	164,263..164,718	456	<=
190.	hypothetical protein	164,814..165,311	498	<=
191.	hypothetical protein	165,313..165,981	669	<=
192.	hypothetical protein	165,978..166,397	420	<=
193.	hypothetical protein	166,407..166,838	432	<=
194.	hypothetical protein	166,847..167,470	624	<=
195.	hypothetical protein	167,472..168,371	900	<=
196.	hypothetical protein	168,358..168,924	567	<=
197.	hypothetical protein	168,947..169,921	975	<=
198.	hypothetical protein	169,923..171,023	1101	<=
199.	hypothetical protein	171,020..171,340	321	<=
200.	hypothetical protein	171,337..171,798	462	<=
201.	hypothetical protein	171,867..173,735	1869	<=
202.	hypothetical protein	173,737..174,030	294	<=
203.	hypothetical protein	174,083..174,835	753	<=
204.	hypothetical protein	174,875..175,993	1119	<=
205.	hypothetical protein	176,056..176,763	708	<=
206.	acyl carrier protein	176,804..177,145	342	<=
207.	hypothetical protein	177,204..177,572	369	<=
208.	hypothetical protein	177,685..178,737	1053	<=
209.	hypothetical protein	178,762..179,058	297	<=
210.	hypothetical protein	179,067..179,357	291	<=
211.	hypothetical protein	179,367..179,927	561	<=
212.	hypothetical protein	180,306..180,758	453	<=
213.	putative N-acetyltransferase	180,768..181,250	483	<=
214.	UvsX-like recombinase	181,330..183,780	2451	<=
215.	single strand DNA binding protein	183,828..185,231	1404	<=
216.	RNA polymerase sigma factor	185,253..186,320	1068	<=
217.	hypothetical protein	186,320..187,201	882	<=
218.	hypothetical protein	187,191..187,664	474	<=
219.	hypothetical protein	187,772..187,930	159	<=
220.	SWIM-type domain-containing protein	187,981..188,565	585	<=
221.	hypothetical protein	188,558..188,938	381	<=
222.	hypothetical protein	189,187..189,678	492	<=
223.	ATP-dependent helicase	189,675..190,799	1125	<=

224.	hypothetical protein	190,811..191,065	255	<=
225.	DNA primase	191,147..192,187	1041	=>
226.	hypothetical protein	192,221..193,150	930	<=
227.	hypothetical protein	193,147..193,431	285	<=
228.	hypothetical protein	193,477..194,169	693	<=
229.	hypothetical protein	194,162..194,707	546	<=
230.	holliday junction resolvase	194,736..195,320	585	<=
231.	hypothetical protein	195,360..196,544	1185	=>
232.	hypothetical protein	196,541..196,816	276	=>
233.	hypothetical protein	197,554..198,357	804	=>
234.	peptidase	198,341..199,351	1011	=>
235.	hypothetical protein	199,357..199,728	372	=>
236.	hypothetical protein	200,102..200,224	123	<=
237.	hypothetical protein	200,621..201,634	1014	=>
238.	DNA polymerase III subunit gamma	201,685..202,836	1152	=>
239.	exonuclease SbcC	202,850..205,177	2328	=>
240.	putative DNA-binding protein	205,330..205,470	141	=>
241.	hypothetical protein	205,467..205,901	435	=>
242.	virion structural protein	205,898..206,614	717	=>
243.	hypothetical protein	206,614..207,054	441	=>
244.	hypothetical protein	207,054..207,938	885	=>
245.	hypothetical protein	207,948..208,388	441	=>
246.	hypothetical protein	208,404..209,174	771	=>
247.	hypothetical protein	209,267..209,818	552	=>
248.	recombination-related endonuclease	209,818..210,882	1065	=>
249.	hypothetical protein	211,003..211,692	690	=>
250.	virion structural protein	211,854..213,170	1317	=>
251.	virion structural protein	213,183..214,307	1125	=>
252.	head-tail connector protein	214,294..215,475	1182	=>
253.	virion structural protein	215,489..216,295	807	=>
254.	hypothetical protein	216,346..217,041	696	=>
255.	HNH endonuclease	217,044..217,424	381	=>
256.	Virion structural protein	217,424..218,230	807	=>
257.	hypothetical protein	218,230..218,754	525	=>
258.	DNA helicase	218,754..225,041	6288	=>
259.	lysozyme	225,084..225,557	474	=>
260.	DNA polymerase I	225,640..228,768	3129	=>
261.	hypothetical protein	228,814..229,140	327	=>
262.	DNA polymerase III, beta subunit	229,118..230,314	1197	=>
263.	hypothetical protein	230,324..230,767	444	=>
264.	recombination protein	230,767..231,315	549	=>
265.	DNA repair helicase	231,317..232,894	1578	=>
266.	putative DNA exonuclease	232,933..233,556	624	=>
267.	hypothetical protein	233,560..234,240	681	=>
268.	hypothetical protein	234,254..234,463	210	=>
269.	DNA methyltransferase	234,460..235,311	852	=>
270.	class I SAM-dependent methyltransferase	235,320..236,411	1092	=>
271.	hypothetical protein	236,424..236,903	480	=>
272.	DNA polymerase III subunit 3'-5' exoribonuclease	236,909..237,907	999	=>
