# Supplementary material for: Novel Giant Phages vB_AerVM_332-Vera and vB_AerVM_332-Igor and Siphophage vB_AerVS_332-Yulya Infecting the Same Aeromonas veronii Strain
Source: Viruses. 2025 Jul 22;17(8):1027. doi: 10.3390/v17081027 (PMC12390700; doi:10.3390/v17081027)
Supplement: Supplementary file 1 [file viruses-17-01027-s001.zip › viruses-3335593-supplementary/Figure S.pdf]

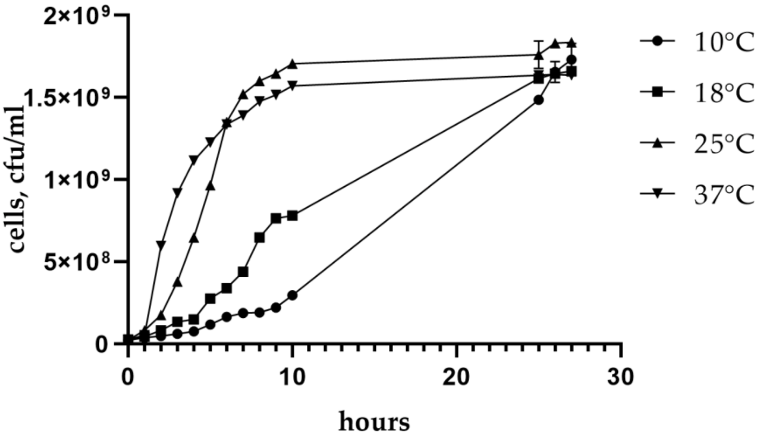

Figure S1. Growth curves for the host strain *A. veronii* CEMTC 7594 at different temperatures. Experiments were performed three times. The bars show standard deviations for each point.

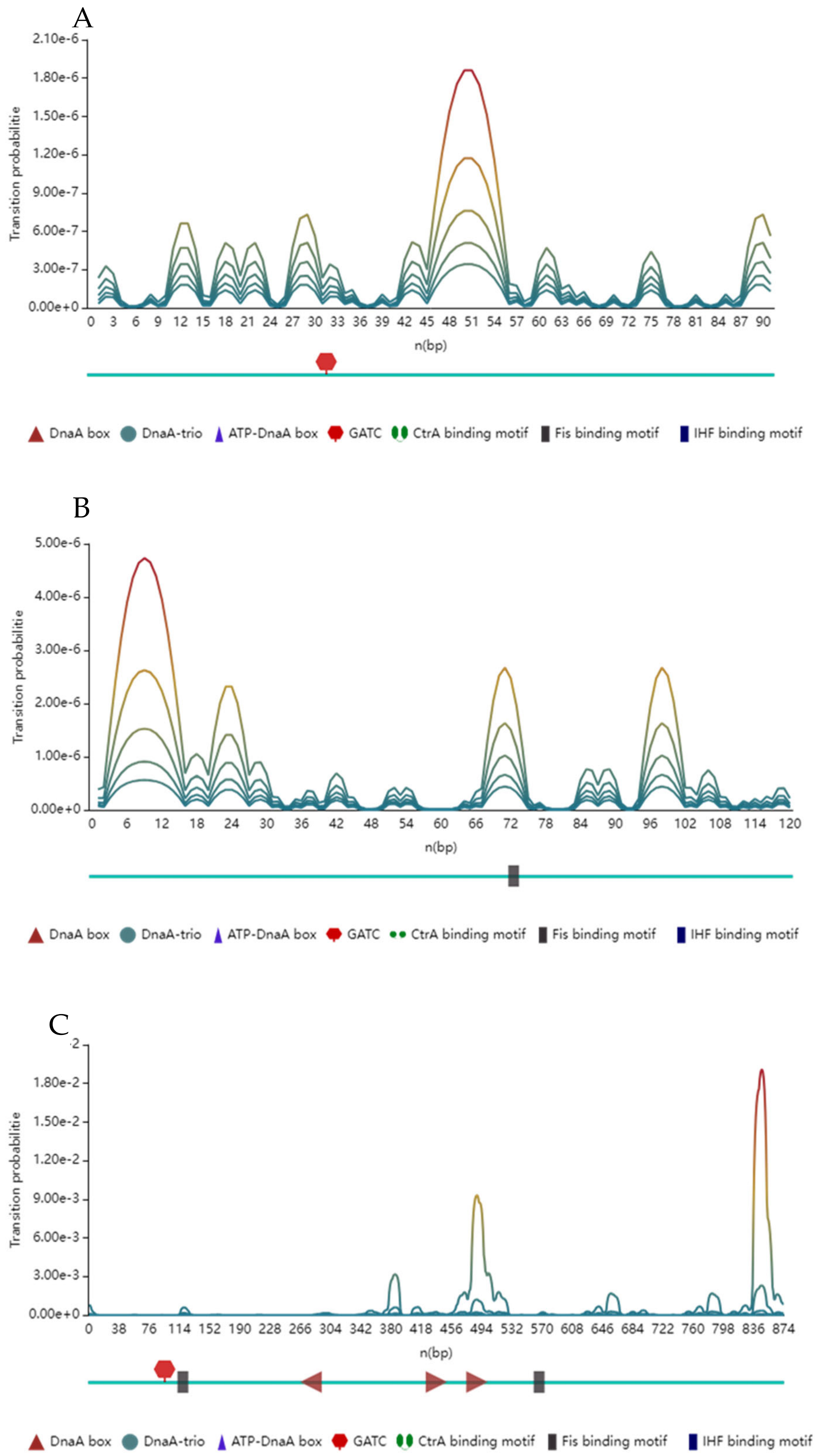

Figure S2. Phage origin prediction by Ori-Finder software. The scheme of the putative origin of replication: (**A**) vB_AerVM_332-Vera genome. (**B**) vB_AerVM_332-Igor genome. (**C**) vB_AerVM_332-Yuliya genome.

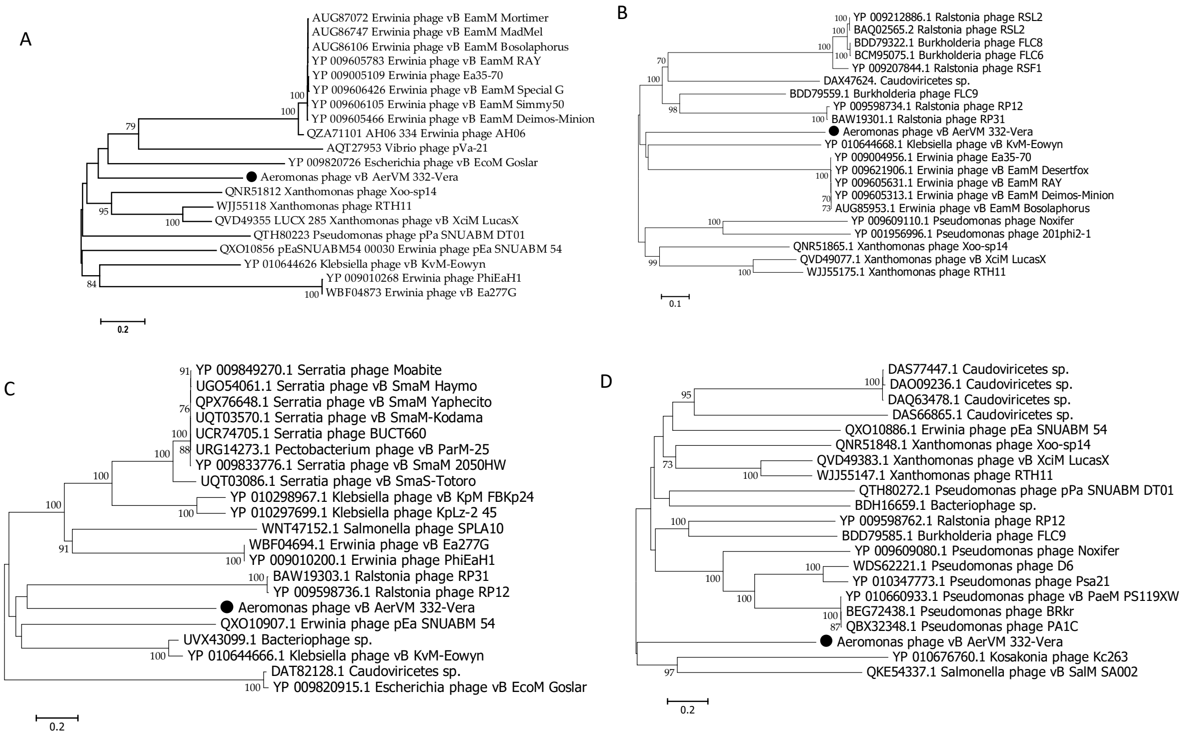

Figure S3. Phylogenetic analysis of vB_AerVM_332-Vera proteins and analogs from other phages. Sequences were aligned using the MAFFT algorithm. Phylogenetic trees were constructed using the maximum likelihood (ML) method based on the JTT matrix-based LG model in MEGA 11.0 with 1000 bootstrap replicates. The studied phage are marked with black circles. A – major capsid protein, B – RNA polymerase beta subunit, C – transglycosylase domain-containing tail fiber protein, D – portal protein.

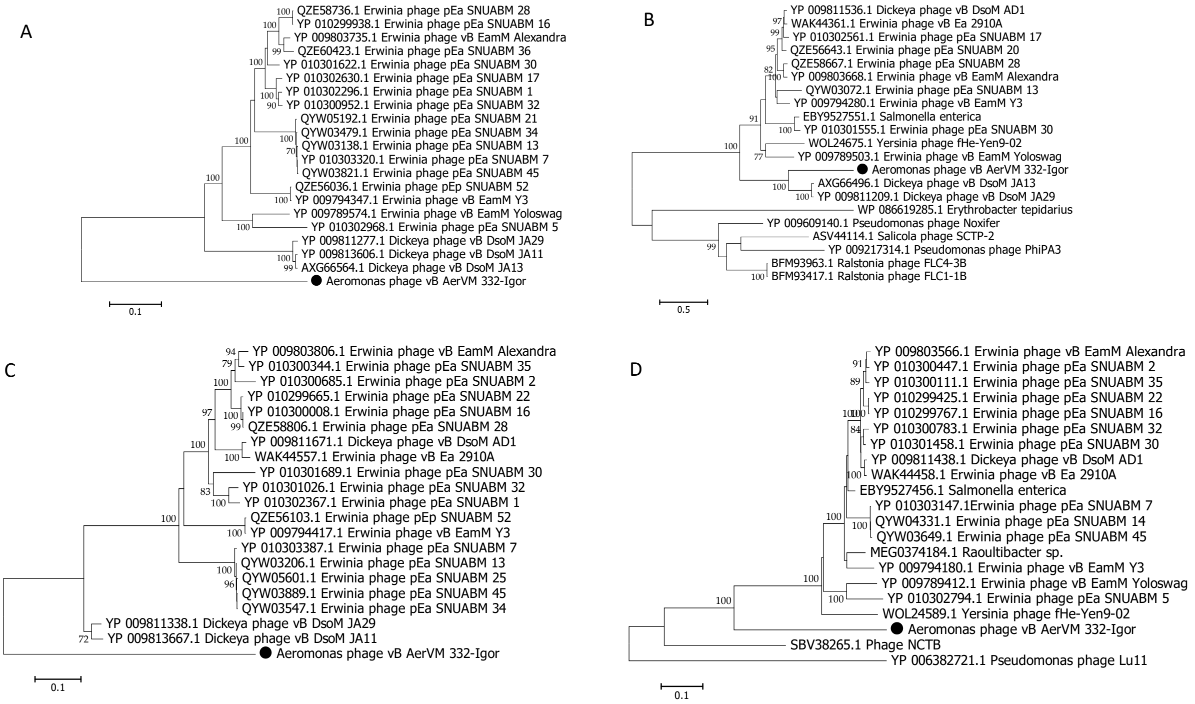

Figure S4. Phylogenetic analysis of vB_AerVM_332-Igor proteins and analogs from other phages. Sequences were aligned using the MAFFT algorithm. Phylogenetic trees were constructed using the maximum likelihood (ML) method based on the JTT matrix-based LG model in MEGA 11.0 with 1000 bootstrap replicates. The studied phage are marked with black circles. A – DNA polymerase, B – tail fiber protein, C – RecA protein, D – portal protein.

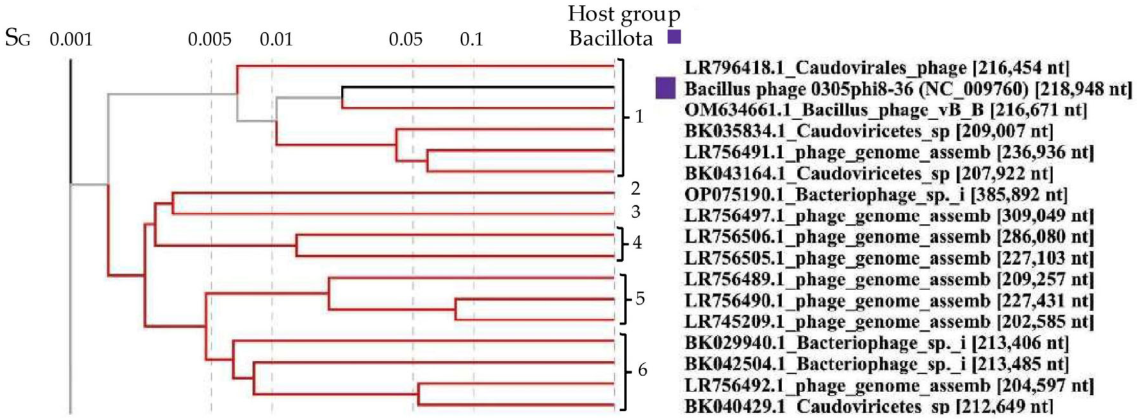

Figure S5. A fragment of the ViPTree dendrogramm showing cluster A (sub-clusters 1-6) of jumbo phages. The numbers indicate the numbers of the sub-clusters.

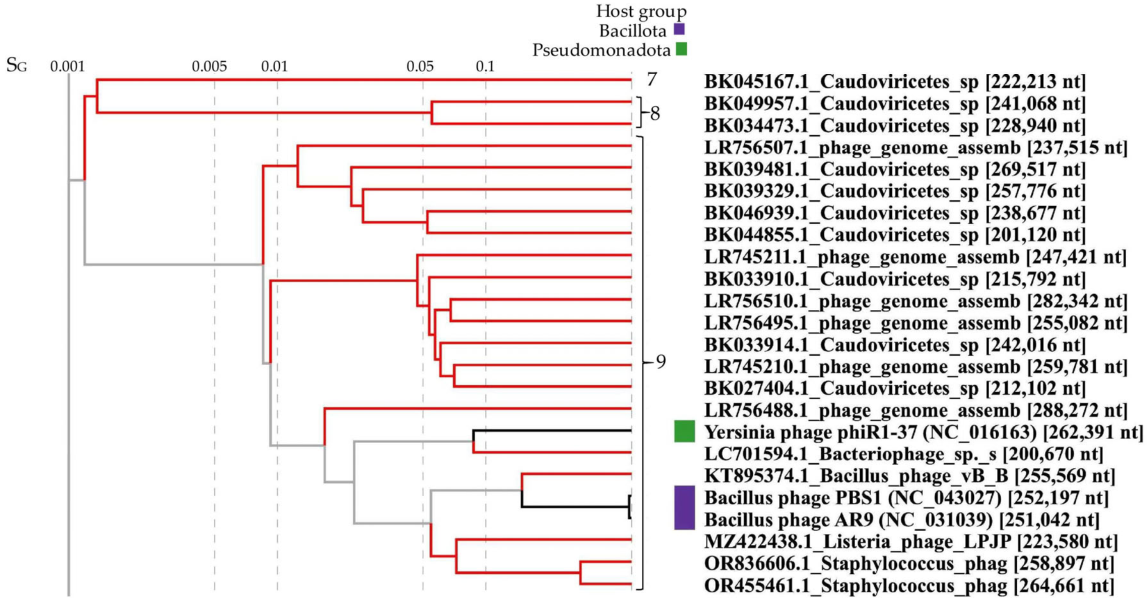

Figure S6. A fragment of the ViPTree dendrogramm showing cluster B (sub-clusters 8-9) of jumbo phages. The numbers indicate the numbers of the sub-clusters.

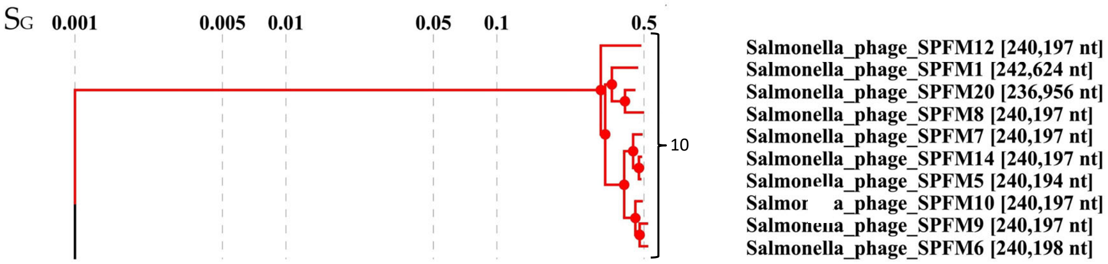

Figure S7. A fragment of the ViPTree dendrogramm showing cluster A (sub-cluster 10) of jumbo phages. The number indicate the number of the sub-clusters.

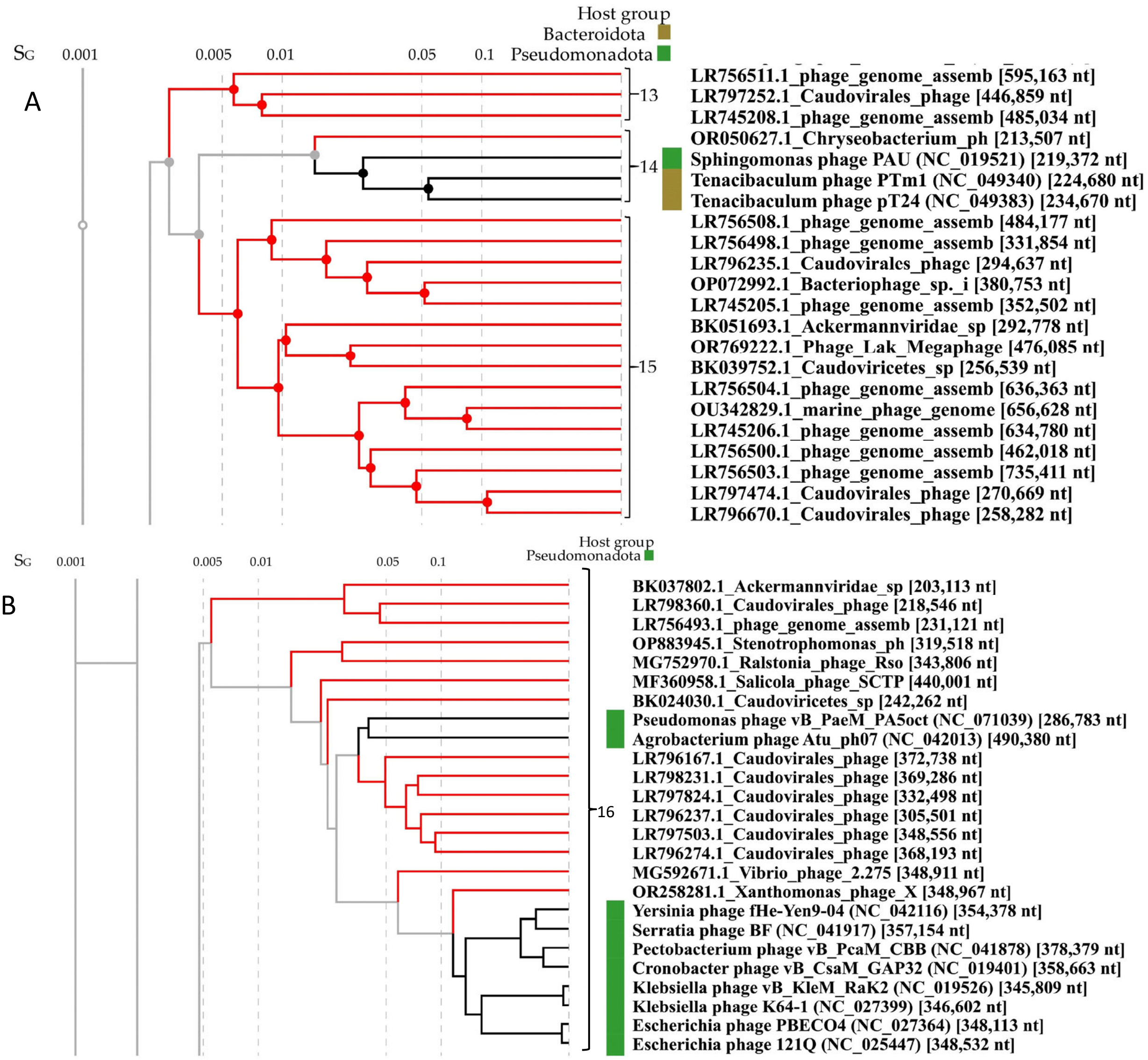
Host group
Bacteroidota
Pseudomonadota
SG
0.001
0.005
0.01
0.05
0.1
A
13
14
15
LR756511.1_phage_genome_assemb [595,163 nt]
LR797252.1_Caudovirales_phage [446,859 nt]
LR745208.1_phage_genome_assemb [485,034 nt]
OR050627.1_Chryseobacterium_ph [213,507 nt]
Sphingomonas phage PAU (NC_019521) [219,372 nt]
Tenacibaculum phage PTm1 (NC_049340) [224,680 nt]
Tenacibaculum phage pT24 (NC_049383) [234,670 nt]
LR756508.1_phage_genome_assemb [484,177 nt]
LR756498.1_phage_genome_assemb [331,854 nt]
LR796235.1_Caudovirales_phage [294,637 nt]
OP072992.1_Bacteriophage_sp._i [380,753 nt]
LR745205.1_phage_genome_assemb [352,502 nt]
BK051693.1_Ackermannviridae_sp [292,778 nt]
OR769222.1_Phage_Lak_Megaphage [476,085 nt]
BK039752.1_Caudoviricetes_sp [256,539 nt]
LR756504.1_phage_genome_assemb [636,363 nt]
OU342829.1_marine_phage_genome [656,628 nt]
LR745206.1_phage_genome_assemb [634,780 nt]
LR756500.1_phage_genome_assemb [462,018 nt]
LR756503.1_phage_genome_assemb [735,411 nt]
LR797474.1_Caudovirales_phage [270,669 nt]
LR796670.1_Caudovirales_phage [258,282 nt]
Host group
Pseudomonadota
SG
0.001
0.005
0.01
0.05
0.1
B
16
BK037802.1_Ackermannviridae_sp [203,113 nt]
LR798360.1_Caudovirales_phage [218,546 nt]
LR756493.1_phage_genome_assemb [231,121 nt]
OP883945.1_Stenotrophomonas_ph [319,518 nt]
MG752970.1_Ralstonia_phage_Rso [343,806 nt]
MF360958.1_Salicola_phage_SCTP [440,001 nt]
BK024030.1_Caudoviricetes_sp [242,262 nt]
Pseudomonas phage vB_PaeM_PA5oct (NC_071039) [286,783 nt]
Agrobacterium phage Atu_ph07 (NC_042013) [490,380 nt]
LR796167.1_Caudovirales_phage [372,738 nt]
LR798231.1_Caudovirales_phage [369,286 nt]
LR797824.1_Caudovirales_phage [332,498 nt]
LR796237.1_Caudovirales_phage [305,501 nt]
LR797503.1_Caudovirales_phage [348,556 nt]
LR796274.1_Caudovirales_phage [368,193 nt]
MG592671.1_Vibrio_phage_2.275 [348,911 nt]
OR258281.1_Xanthomonas_phage_X [348,967 nt]
Yersinia phage fHe-Yen9-04 (NC_042116) [354,378 nt]
Serratia phage BF (NC_041917) [357,154 nt]
Pectobacterium phage vB_PcaM_CBB (NC_041878) [378,379 nt]
Cronobacter phage vB_CsaM_GAP32 (NC_019401) [358,663 nt]
Klebsiella phage vB_KleM_RaK2 (NC_019526) [345,809 nt]
Klebsiella phage K64-1 (NC_027399) [346,602 nt]
Escherichia phage PBECO4 (NC_027364) [348,113 nt]
Escherichia phage 121Q (NC_025447) [348,532 nt]

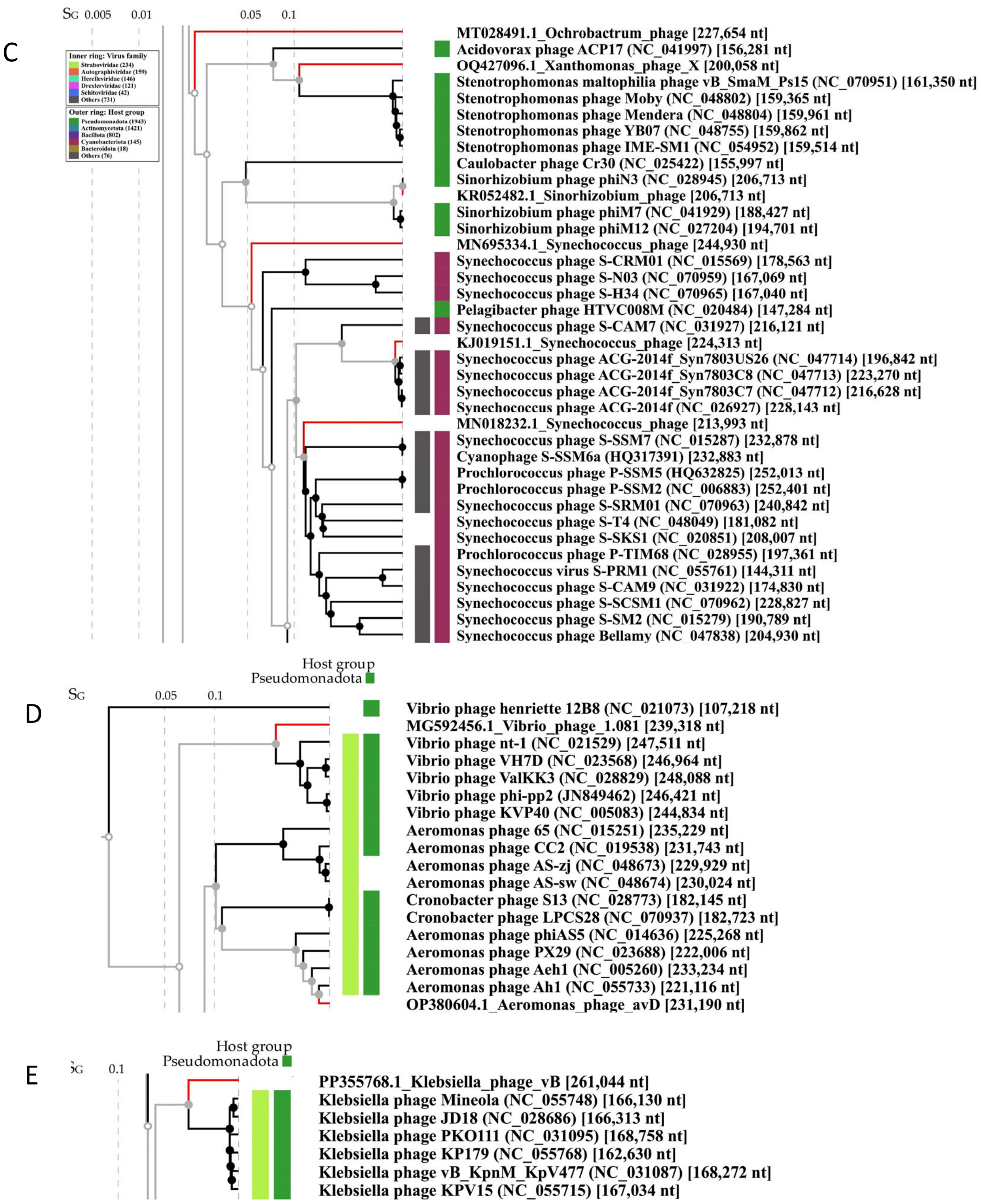

Figure S8. A fragment of the ViPTree dendrogramm showing cluster F of jumbo phages. A – sub-clusters 13, 14 and 15; B – sub-cluster 16; C, D and E – sub-cluster 17.

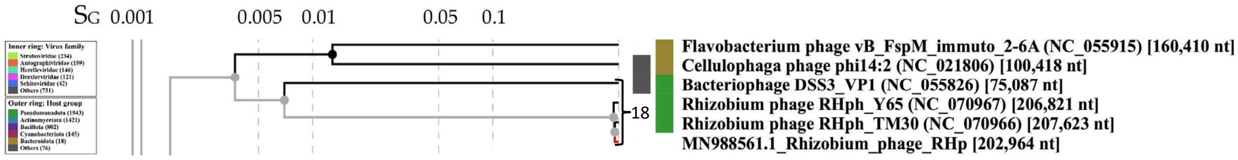

Figure S9. A fragment of the ViPTree dendrogramm showing cluster F of jumbo phages (sub-cluster 18).

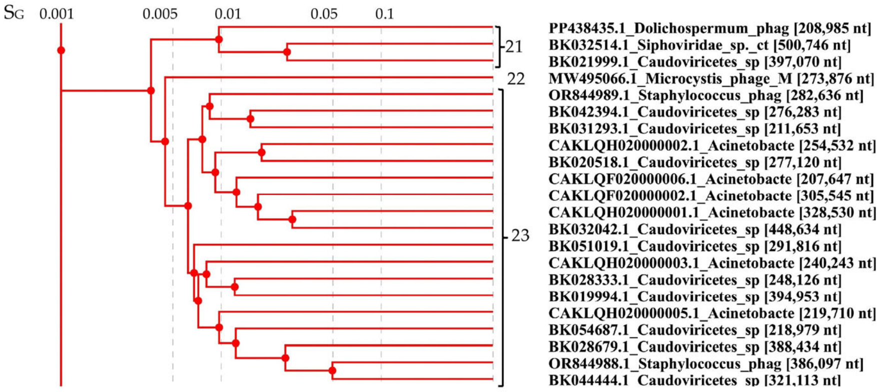

Figure S10. A fragment of the ViPTree dendrogramm showing cluster I (sub-clusters 21-23) of jumbo phages. The numbers indicate the numbers of the sub-clusters.

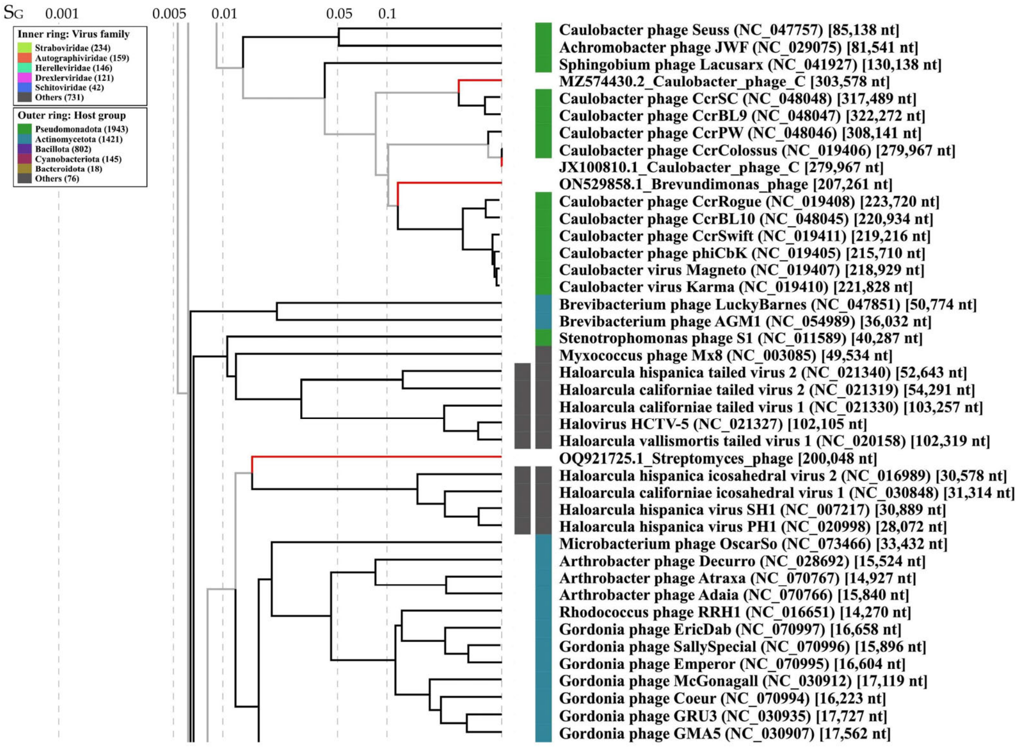

Figure S11. A fragment of the ViPTree dendrogramm showing cluster J (a fragment of sub-clusters 24) of jumbo phages.
