# Supplementary material for: Novel Giant Phages vB_AerVM_332-Vera and vB_AerVM_332-Igor and Siphophage vB_AerVS_332-Yulya Infecting the Same Aeromonas veronii Strain
Source: Viruses. 2025 Jul 22;17(8):1027. doi: 10.3390/v17081027 (PMC12390700; doi:10.3390/v17081027)
Supplement: Supplementary file 1 [file viruses-17-01027-s001.zip › viruses-3335593-supplementary/Table S1 final.pdf]

Table S1. ***Aeromonas*** **strains screened in host range assay**

№	CEMTC* number	Collection date	Material	Source	GenBank Accession
			Aeromonas veronii		
1	1354	Jun 2012	bottom silt	Altai territory, Lake Teply Klyuch	OP849653
2	1362	Jun 2012	fresh water	Altai territory, Lake Teply Klyuch	ON479597
3	1369	Jun 2012	bottom silt	Altai territory, Lake Teply Klyuch	OP218042
4	1414	Sep 2012	thermal water	Kamchatka, Uzon Caldera	OP849655
5	1416	Sep 2012	sediments	Kamchatka, Uzon Caldera	OP218043
6	4064	Aug 2020	fresh water	Novosibirsk region, Inya River	ON479603
7	5502	May 2022	fresh water	Altai Republic, Maly Ilgumen River	OQ332355
8	5892	May 2022	fresh water	Altai territory, Itkul River	OQ332356
9	6243	Jun 2022	fresh water	Tatarstan, Bulak River	OQ332357
10	6289	Jul 2022	spring water	Kemerovo region, spring	OQ332359
11	6436	Jun 2022	fresh water	Tatarstan, Lake Nizhny Kaban	OP659006
12	6618	Jul 2022	fresh water	Novosibirsk region, pond	OP659007
13	7112	Aug 2022	fresh water	Tatarstan, Kazanka River	OQ913692
14	7445	Jul 2022	fresh water	Krasnodar region, Sochi, pond	OQ332362
15	7500	Aug 2022	swamp water	Novosibirsk region, forest swamp	OQ913693
16	7594**	Jul 2022	water+sediments	Krasnodar territory, Sochi, pond	OR226262
17	7638	May 2022	fresh water	Novosibirsk region, Lake Bolshoe Kamyshovoe	OQ913694
18	8132	Dec 2022	fresh water	Krasnodar region, Sochi, pond	OQ913696
19	9509	Mar 2023	roadside snow	Novosibirsk	PP499232
20	9558	Jul 2023	fresh water	North Kazakhstan region, Lake Pestroye	PP660207
21	9589	Jul 2023	salt water	North Kazakhstan region, Lake Solenoe	PP499233
22	9698	Aug 2023	fresh water	Kyrgyzstan, Sary-Chelek Biosphere Reserve, Lake Irikel	PP499234
23	10580	Aug 2024	fresh water	Altai Republic, Lake Teletskoye	PQ569580
			Aeromonas caviae		
24	9512	Aug 2023	rainwater	Novosibirsk, storm sewer	PP499219
25	9579	Jul 2023	fresh water	North Kazakhstan region, Lake	
			Aeromonas hydrophila		
26	2094	Feb 2015	fresh water	Vietnam, Dalat	OP849657
27	6279	Jul 2022	fresh water	Kemerovo region, Lake Aprelka	OQ332358
28	7426	Aug 2022	fresh water	Primorsky territory, Lazovsky District, pond	OQ332361
29	8139	Dec 2022	fresh water	Krasnodar territory, Sochi, pond	OQ834572
30	9278	May 2023	hospital bathroom	Kazakhstan, Ust-Kamenogorsk, hospital	PP499221
31	10598	Aug 2024	puddle water	Novosibirsk region, solid municipal waste landfill	PQ569569
			Aeromonas jandaei		
32	1458	Dec 2012	fresh water	India, North Goa, Dudhsagar Falls	ON479599
			Aeromonas media		
33	5475	May 2022	fresh water	Novosibirsk region, Krutikha River	OP659002
34	7654	May 2022	fresh water	Novosibirsk region, Oesh River	OQ332364
35	8112	Dec 2022	fresh water	Krasnodar territory, Sochi, pond	OQ834573
36	8147	Oct 2022	fresh water	Omsk, Irtysh River	OQ913697
37	8204	Oct 2022	fresh water	Omsk, Irtysh River	PQ569570
38	9051	Aug 2022	fresh water	Novosibirsk region, Koen River	PQ569571
39	9069	Aug 2022	fresh water	Novosibirsk region, Big Koen River	PP499222
			Aeromonas popoffii		
40	1430	Sep 2012	fresh water	Kamchatka, Death Valley, river	ON479598
41	3381	May 2018	fresh water	Altai Republic, Lake Geysernoye	ON479601
42	4062	Aug 2020	fresh water	Novosibirsk region, Inya River	ON479602
			Aeromonas rivipollensis		

43	6434	Jun 2022	fresh water	Tatarstan, Lake Nizhny Kaban	OP659005
44	8700	Oct 2022	fresh water	Saratov region, Yeruslan River	PP660206
45	9040	Aug 2022	fresh water	Novosibirsk region, Koen River	PP499223
46	9047	Aug 2022	fresh water	Altai Republic, Koksu River	PP499224
47	9166	Mar 2023	thermal water	Buryatia, Khakusy	PP499225
Aeromonas rivuli					
48	8512	Oct 2022	fresh water	Krasnodar territory, Leningradskaya village, pond	OR226263
***Aeromonas salmonicid*a**					
49	3364	May 2018	fresh water	Altai Republic, Lake Geysernoye	ON479600
50	3375	May 2018	fresh water	Altai Republic, Lake Geysernoye	OR226260
51	4331	Oct 2021	fresh water	Novosibirsk region, Lake Uryupino	OP849658
52	4537	Oct 2021	fresh water	Novosibirsk region, pond	OP218048
53	4546	Aug 2021	thermal water	Sakhalin region, Ogonki village	ON479605
54	5456	May 2022	fresh water	Altai Republic, Lake Geysernoye	OP218051
55	5599	Oct 2021	fresh water	Novosibirsk region, pond	OP849659
56	5828	May 2022	swamp water	Altai territory, Sokolovo village	OP849660
57	5875	May 2022	fresh water	Altai territory, Sokolovo village, Itkul River	OR226261
58	5885	May 2022	spring water	Kemerovo region, Kazansky spring	
59	6952	Aug 2022	fresh water	Tatarstan, Lake Protochnoye	
60	7512	Aug 2022	swamp water	Novosibirsk region, forest swamp	OQ332363
61	7722	Oct 2022	fresh water	Novosibirsk, Zyryanka River	OQ913695
62	8183	Oct 2022	river bank soil	Omsk, Irtysh River	OQ913698
63	8504	Oct 2022	river bank soil	Novosibirsk region, Lake South	PQ569572
64	9317	May 2023	fresh water	Novosibirsk region, Lake Nekrasovskoye	PP499226
65	9349	May 2023	fresh water	Novosibirsk region, Lake Kazennoe	PP499227
66	9353	May 2023	fresh water	Novosibirsk region, Lake Kamnevo	PQ569573
67	9460	May 2023	fresh water	Saint Petersburg, Udelny Park, stream	PQ569574
68	9521	Jul 2023	fresh water	Altai Republic, Ukok plateau, Lake Gusinoe	PQ569575
69	9703	Aug 2022	fresh water	Kyrgyzstan, Chatkal Range, Kara-Suu River	
70	9763	Aug 2022	fresh water	Kyrgyzstan, Padysh-Ata Nature Reserve, Padysh-Ata River	PP499229
71	10144	Mar 2023	fresh water	Donetsk, Starobeshevskoe reservoir	PQ569576
72	10555	Jul 2024	fresh water	Sakhalin, Yuzhno-Sakhalinsk, Lake Verkhnee	PQ569577
73	10587	Aug 2024	fresh water	Altai Republic, Big Chili River	PQ569578
74	10592	Aug 2024	fresh water	Altai Republic, Koldor River	PQ569579
Aeromonas sobria					
75	5646	May 2022	fresh water	Altai Republic, Lake Geysernoye	
76	9695	Aug 2023	fresh water	Kyrgyzstan, Sary-Chelek Biosphere Reserve, Lake Sary-Chelek	PP499231
***Aeromonas* sp.**					
77	4529	Oct 2021	fresh water	Novosibirsk, pond	OP218047

*– Collection of Extremophile Microorganisms and Type Cultures (CEMTC) of ICBFM SB RAS; ** - host strain
